# Characterisation of sterol biosynthesis and validation of 14α**-**demethylase as a drug target in *Acanthamoeba*

**DOI:** 10.1038/s41598-017-07495-z

**Published:** 2017-08-15

**Authors:** Scott Thomson, Christopher A. Rice, Tong Zhang, RuAngelie Edrada-Ebel, Fiona L. Henriquez, Craig W. Roberts

**Affiliations:** 1 Strathclyde Institute of Pharmacy and Biomedical Sciences, University of Strathclyde, Glasgow, G4 0RE UK; 2 Institute of Biomedical and Environmental Health Research, School of Science and Sport, University of West of Scotland, Paisley, PA1 2BE UK; 3 Center for Tropical and Emerging Global Diseases, University of Georgia, Athens, Georgia 30602 USA

## Abstract

The soil amoebae *Acanthamoeba* causes *Acanthamoeba* keratitis, a severe sight-threatening infection of the eye and the almost universally fatal granulomatous amoebic encephalitis. More effective treatments are required. Sterol biosynthesis has been effectively targeted in numerous fungi using azole compounds that inhibit the cytochrome P450 enzyme sterol 14α-demethylase. Herein, using Gas Chromatography Mass Spectrometry (GCMS), we demonstrate that the major sterol of *Acanthamoeba castellanii* is ergosterol and identify novel putative precursors and intermediate sterols in its production. Unlike previously reported, we find no evidence of 7-dehydrostigmasterol or any other phytosterol in *Acanthamoeba*. Of five azoles tested, we demonstrate that tioconazole and voriconazole have the greatest overall inhibition for all isolates of *Acanthamoeba castellanii* and *Acanthamoeba polyphaga* tested. While miconazole and sulconazole have intermediate activity econazole is least effective. Through GCMS, we demonstrate that voriconazole inhibits 14α**-**demethylase as treatment inhibits the production of ergosterol, but results in the accumulation of the lanosterol substrate. These data provide the most complete description of sterol metabolism in *Acanthamoeba*, provide a putative framework for their further study and validate 14α**-**demethylase as the target for azoles in *Acanthamoeba*.

## Introduction

*Acanthamoeba* is a genus of normally free-living amoeba found in the environment throughout the world. Amoebozoa are single celled organisms and are generally believed to be phylogenetically close to fungi and metazoans, together forming the Amorphea^1^. *Acanthamoeba* are mixotrophic and have the ability to encyst when the environment becomes hostile. *Acanthamoeba* can be a facultative pathogen with the ability to cause serious infections. The most common of these is *Acanthamoeba* keratitis (AK) that can lead to corneal damage and eventual blindness. Clinical cases of AK are most commonly found in contact lens wearers or following trauma to the eye. It is estimated that AK occurs in 1 in 30,000 contact lens wearers in the UK^2^. *Acanthamoeba* can also be an opportunistic pathogen causing granulomatous amoebic encephalitis (GAE) in people with suppressed immune systems.

Treatment of AK is currently arduous and extremely difficult for the patient requiring hospitalisation due to the requirement for alternating eye drops containing either propamidine isethionate and polyhexamethyl biguanide, or propamidine isethionate and neomycin to be applied on an hourly basis for prolonged periods. To compound matters resistance to polyhexamethyl biguanide is developing^3, 4^. An additional problem is that many drugs do not work so well against cysts or even induce *Acanthamoeba* to encyst. GAE is almost always fatal despite extreme measures including multiple drug combinations combined with surgical procedures or cryotherapy. A potential exploitable difference between *Acanthamoeba* and their human host is sterol metabolism. While the principal membrane sterol in humans is cholesterol, previous studies have reported that ergosterol and 7-dehydrostigmasterol as the major sterols of *Acanthamoeba* ^5^. Furthermore, sterol metabolism has been successfully targeted to treat a number of fungal infections of humans. Notably, a number of azole antifungal drugs are known to curtail sterol production through the inhibition of sterol 14α**-**demethylase. While this would have the obvious effect of reducing the integrity of *Acanthamoeba* membranes alternative mechanisms of action include the build up of toxic precursors of ergosterol. It is encouraging that a number of azoles designed to target fungal sterol 14α**-**demethylase have proven effective in treatment of patients with *Acanthamoeba* infections^6–9^.

This study was initiated to characterise the sterol composition of *Acanthamoeba* and correlate this with information in the transcriptome project to construct a scheme for sterol biosynthesis in this organism as well as determine the susceptibilities of a number of *A*. *castellanii* and *A*. *polyphaga* strains to a variety of azoles (econazole, miconazole, sulconazole, tioconazole and voriconazole) using a previously described AlamarBlue assay^10, 11^. We demonstrate that ergosterol as previously reported is the major sterol of *Acanthamoeba*, but find no evidence of 7-dehydrostigmasterol as previously reported^5^ and through the use of GCMS provide the first direct evidence that voriconazole inhibits 14α**-**demethylase in *Acanthamoeba*.

## Results

### Ergosterol is the major sterol of *Acanthamoeba*

**Figure 1:**
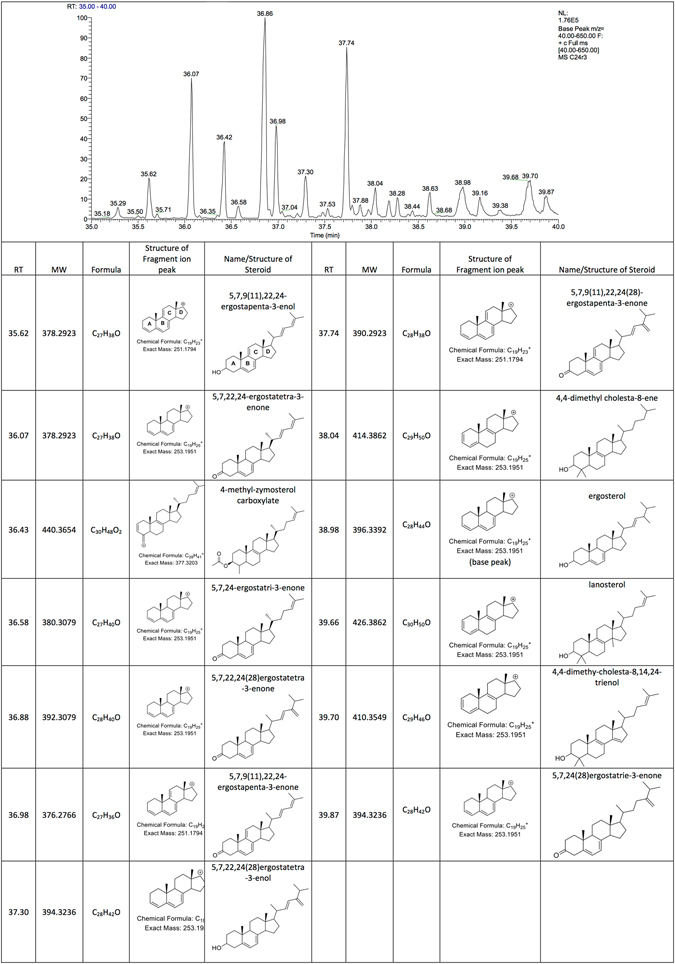
Ion chromatogram demonstrating the principal sterols of *Acanthamoeba* (clinical T4 strain), their retention times (RT), molecular weights (MW) with observed base peaks, chemical formulae, names and structures. The structures on column 4 represent the ‘fragment ion peak’, while the structure on column 5 represent the ‘molecular ion peak’. The fragment ion peak is the base peak which confirms the positions of the double bonds on rings B and C, this information is combined with the molecular ion peak to elucidate the complete structures.

GCMS was used to determine the sterol composition of *A*. *castellanii* (Neff strain) and a T4 clinical strain. Ergosterol was the major sterol product detected in both strains of *A*. *castellanii*. We found no evidence of cholesterol, demosterol, campesterol, stigmasterol or 7-dehydrostigmasterol in either strain (Fig. 1 and Supplementary data). We found a number of canonical ergosterol precursors including lanosterol, 4,4-dimethyl-cholesta-8,14,24-trienol, 4-methyl-zymosterol carboxylate, 5,7,24(28)ergostatrie-3-enol and 5,7,22,24(28)ergostatetra-3-enol. We did not detect 14-demethyl lanosterol, however 4,4 dimethyl cholesta-8-ene, which is a likely reversible intermediate derivative of 14-demethyl lanosterol was readily detected. The interconversion between 14-demethyl lanosterol and 4,4 dimethyl cholesta-8-ene can occur by simultaneous dehydrogenation (oxidation) and reduction at C-24 and C-25 olefinic bond. We did not detect 3-keto-4-methyl-zymosterol, 4-methyl-zymosterol, zymosterol, fecosterol or episterol. However, we detected a number of non-canonical sterols suggesting that sterol biosynthesis occurs in *Acanthamoeba* by a modified alternative putative pathway that involves the sequential demethylation and desaturation of lanosterol to 5,7,9(11),22,24-ergostapenta-3-enol, which is then oxidised to 5,7,9(11),22,24-ergostapenta-3-enone (candidate enzymes are ELR12281 and ELR22222, respectively). This would then be reduced to 5,7,22,24(28)-ergostatetra-3-enone (3 possible candidate enzymes are ELR17146, ELR23035 or ELR14820) by losing the 9(11) double bond then converted to 4-methylzymosterol carboxylate via a C-4 methyl sterol oxidase (candidate ELR23089) or to zymosterol via a reductase (candidate enzyme ELR23089).

**Figure 2:**
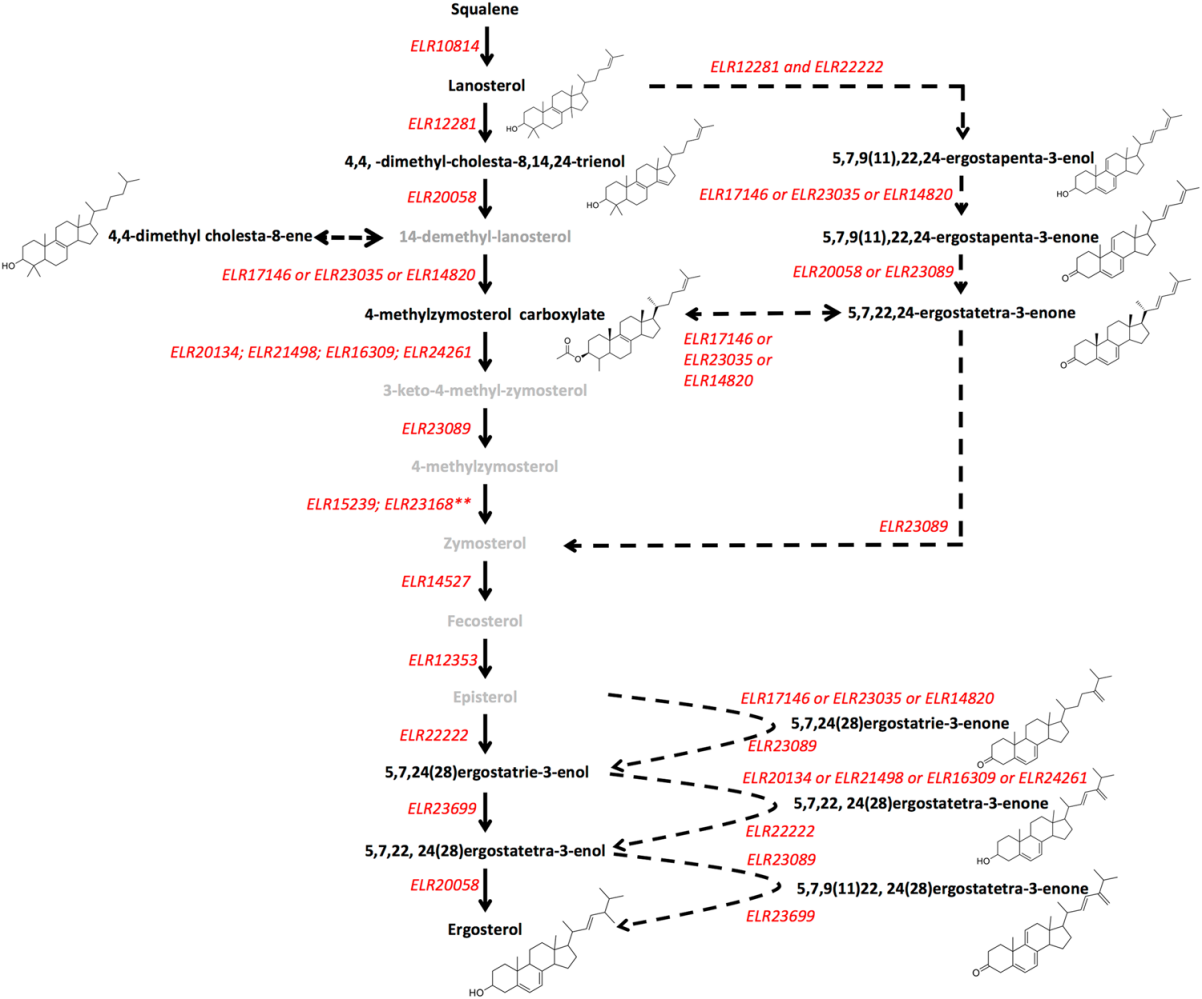
Proposed scheme of sterol biosynthesis in *Acanthamoeba*. Products and intermediates in black were identified by GCMS while those in grey were not. Candidate enzymes, named according to their GenBank Accession numbers, are annotated in red. **Denotes that this protein is not an enzyme but an anchor protein.

We also detected 5,7,24(28)-ergostatrie-3-enone, 5,7,22,24(28)-ergostatetra-3-enone and 5,7,9(11),22,24(28)-ergostapenta-3-enone which are oxidised forms of episterol, 5,7,24(28)ergostatrie-3-enol and 5,7,22,24(28)ergostatetra-3-enol, respectively. Again, we have identified candidate enzymes from the *Acanthamoeba* genome for the reversible oxidation (ELR17146 or ELR23035 or ELR14820) and reduction (ELR23089) of these intermediates. The entire proposed scheme is detailed in Fig. 2. The candidate enzymes, their current annotation in the *Acanthamoeb*a transcriptome and the names of their fungal and land plant equivalents are listed in Table 1. PCR and sequencing was used to confirm 14α-demethylase in *A*. *polyphaga* (ATCC 50371), *A*. *castellanii* (ATCC 50370) and *A*. *castellanii* (Neff). These data show no significant differences between the nucleotide or predicted amino acid sequences of the strains examined or with the previously available *A*. *castellanii* (Neff) strain sequences from the transcriptome project (Accession numbers KP970621.1, KP970620.1 and KP970619.1).

**Table 1:** Candidate Enzymes involved in sterol biosynthesis in *Acanthamoeba*.

	Enzyme name	EC	Current annotation in *Acanthamoeba* transcriptome	Confirmation by PCR	Fungal equivalent	Land plant equivalent
ELR10814	Lanosterol synthase	5.4.99.7/5.499.8	(Possible bifunctional) cycloartenol synthase		ERG7	CAS1
ELR12281	Sterol C-14 α demethylase (CYP51)	1.14.13.70	Obtusifoliol 14 α demethylase (CYP51)	*A*. *castellanii* Neff KP970619	ERG11	CYP51G1
				*A*. *polyphaga* 50371 KP970621		
				*A*. *castellanii* 50370 KP970622		
ELR20058	Sterol C-14 reductase	1.3.1.70/1.3.1.71	Similarity 7-dehydrocholesterol reductase	*A*. *castellanii* Neff KP970617	ERG4/24	FK
				*A*. *polyphaga* 50371 KP970618		
ELR17146; ELR23035; ELR14820	C4 methyl sterol oxidase	1.14.13.72	methyl sterol monooxygenase		ERG25	SMO1
ELR20134; ELR21498; ELR16309; ELR24261	Sterol-4α carboxylate 3 dehydrogenase	1.1.1.170	UDP glucose 4 epimerase; 3 beta hydroxysteroid dehydrogenase; NAD dependent epimerase/dehydratase	*A*. *castellanii* Neff KP970622	ERG26	AT3BETAHSD
				*A*. *polyphaga* 50371 KP970623		
ELR23089	3-keto steroid reductase	1.1.1.270	NADPH-dependent carbonyl reductase family protein		ERG27	
ELR15239; ELR23168**	ER anchoring		ERG28; ERG28 family protein		ERG28	
ELR14527	SAM C-24 sterolmethyltransferase	2.1.1.41	sterol 24c-methyltransferase		ERG6	SMT1
ELR12353	C-8 sterol isomerase	5.-.-.-	C-8 sterol isomerase		ERG2	HYD1
ELR22222	C-5 sterol desaturase	1.14.19.20	delta7-sterol 5-desaturase		ERG3	STE1
ELR23699	C-22 sterol desaturase	1.14.14.-	Sterol C22 desaturase-like, putative		ERG5	

### Comparative susceptibilities of *Acanthamoeba castellanii* and *Acanthamoeba polyphaga* isolates to a selection of azoles

**Figure 3:**
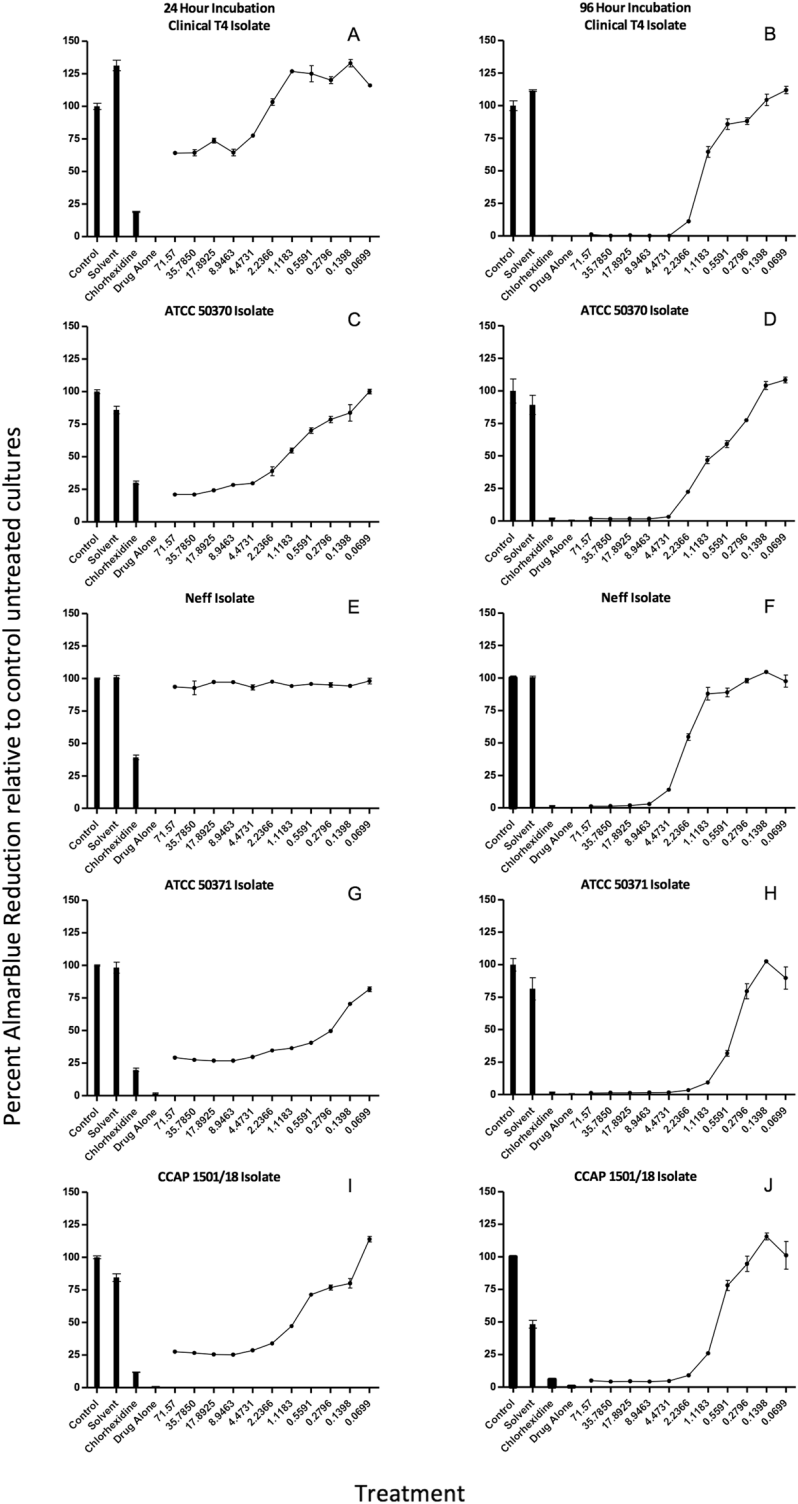
Representative dose response curves, showing the effect of voriconazole on 5 *Acanthamoeba* isolates (clinical T4, ATCC 50370, Neff, ATCC 50371 and CCAP 1501/18).

The growth of 5 different isolates of *Acanthamoeba* species (*A*. *castellanii:* T4 clinical isolate, ATCC 50370, Neff strain; *A*. *polyphaga*: ATCC50371 and CCAP 1501/18) was assessed at 24 hours and 96 hours in the presence of five different azoles (econazole, miconazole, sulconazole, tioconazole and voriconazole), using a modified AlamarBlue microtiter plate assay as described by McBride *et al*.^10^. A representative set of dose response curves, depicting voriconazole treatment of the *Acanthamoeba* isolates is shown in Fig. 3. Data for all azoles tested is shown in Table 2. At 24 hours econazole had no effect on the growth of any of the *Acanthamoeba* tested, whereas miconazole and sulconazole only inhibited ATCC 50370 (IC_50_s 47.7 µM ± 3.5; 46.85 µM ± 6.23, respectively) at 24 hours and tioconazole inhibited ATCC 50370 and ATCC 50371 (IC_50_s 66.66 µM ± 12.12; 8.5 µM ± 1.1, respectively). On the other hand, at 24 hours voriconazole inhibited all isolates with exception of *A*. *castellanii* Neff strain (Fig. 2E). All azoles tested inhibited *Acanthamoeba* growth at 96 hours (Table 2).

**Table 2:** IC_50_ and IC_90_ values for the azoles screened against various *Acanthamoeba* strains at 24 and 96 hours.

	Time:	24 Hours	96 Hours	IC_90_ (µM)	SEM ( ± )	IC_50_ (µM)	SEM ( ± )	IC_90_ (µM)	SEM ( ± )
	Isolate:	IC_50_ (µM)	SEM ( ± )						
	**Clinical T4**	*	—	*	—	*	—	*	—
	**ATCC 50370**	*	—	*	—	90.64	22.87	*	—
	**ATCC 50371**	*	—	*	—	*	—	*	—
	**Neff**	*	—	*	—	140.27	8.61	197.14	28.28
	**CCAP 1501/18**	*	—	*	—	104.39	18.9	274.1	16.29
	**Clinical T4**	*	—	*	—	*	—	*	—
	**ATCC 50370**	*	—	*	—	8.28	0.12	*	—
	**ATCC 50371**	*	—	*	—	21.17	6.05	33.4	6.99
	**Neff**	*	—	*	—	*	—	*	—
	**CCAP 1501/18**	*	—	*	—	13.42	3.31	28.25	0.7
	**Clinical T4**	*	—	*	—	24.43	5.07	40.97	4.91
	**ATCC 50370**	*	—	*	—	11.48	3.58	47.13	27.44
	**ATCC 50371**	5.53	3.45	18.36	7.43	15.09	11.81	21.36	14.71
	**Neff**	*	—	*	—	15.13	6.58	23.79	6.77
	**CCAP 1501/18**	*	—	*	—	4.70	0.64	34.06	23.84
	**Clinical T4**	30.84	18.5	102.22	9.05	13.04	3.94	76.05	37.41
	**ATCC 50370**	12.10	6.16	*	—	3.43	0.12	18.72	2.26
	**ATCC 50371**	5.57	1.75	42.66	6.87	5.38	2.94	30.13	23.99
	**Neff**	*	—	*	—	8.23	0.44	18.55	2.89
	**CCAP 1501/18**	*	—	*	—	4.63	0.84	18.46	6.41
	**Clinical T4**	*	—	*	—	1.91	0.43	7.57	1.54
	**ATCC 50370**	1.05	0.069	18.27	0.96	0.54	0.001	2.46	0.08
	**ATCC 50371**	0.64	0.36	4.6	3.11	0.98	0.57	1.68	0.8
	**Neff**	*	—	*	—	2.1	0.03	4.46	1.15
	**CCAP 1501/18**	0.82	0.24	3.28	0.26	0.92	0.16	2.15	0.5

*IC_50_ or IC_90_ value was not consistently obtained within the range of concentrations tested.

### GCMS demonstrates voriconazole specifically inhibits 14-α -demethylase

**Figure 4:**
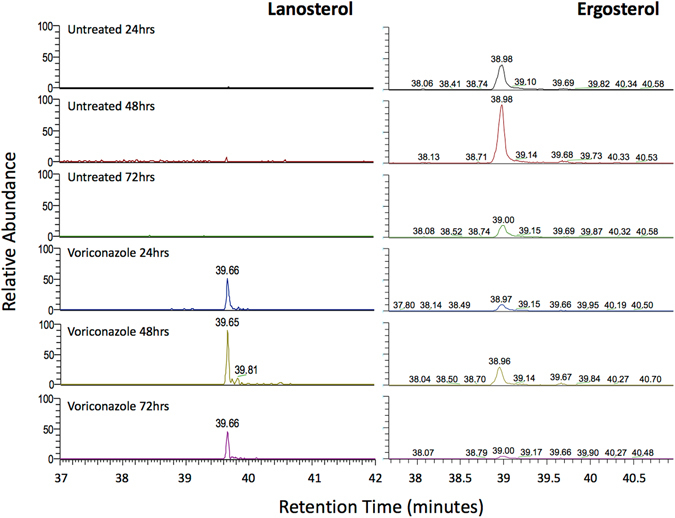
Extracted ion chromatograms for lanosterol (*m/z* = 426.38, RT = 39.66 min) and ergosterol (*m/z* = 396.33, RT = 38.98 min) at 24, 48, and 72 hours before and after treatment with voriconazole.

As the drug assay demonstrated that voriconazole was the most effective of the drugs tested, being shown to be capable of inducing actual cell death as compared to stasis its method of action was confirmed via GCMS. Lanosterol was undetectable in untreated *Acanthamoeba* at all time points examined, but accumulated to detectable levels cells treated with voriconazole at concentrations equivalent to a concentration that inhibited 50% of *Acanthamoeba* growth (IC_50_) (Fig. 4). In contrast 4,4-dimethyl-cholesta-8,14,24-trienol, a downstream product of 14α**-**demethylase was abundantly detected in non-treated control cultures, but not voriconazole-treated cultures (data not shown). Ergosterol levels were reduced in voriconazole treated cultures relative to control cultures at all time points examined (Fig. 4). These results therefore demonstrate that voriconazole inhibits *Acanthamoeba* 14α**-**demethylase and results in inhibition of ergosterol production.

## Discussion

Treatments for diseases caused by *Acanthamoeba* are far from ideal and rely on a combination of drugs that have shown some efficacy normally in historical or recent clinical cases. In cases where chlorhexidine resistant *Acanthamoeba* have been encountered topical voriconazole has been used^7^. There have also been case reports of successful treatment of AK and GAE patients using orally administered voriconazole^6–9, 12^. The choice of azole used in each of these cases is not based on laboratory or clinical controlled studies, but rather some positive outcomes. Clearly laboratory studies have the ability to inform clinical studies and have the potential to improve treatments. Previous studies have examined relatively few azoles against relatively few isolates of *Acanthamoeba* making direct comparisons and generalisations regarding efficacy difficult^13, 14^. The situation is further compounded by the use of diverse laboratory assay systems previously in common use. Stepping back from these empirical studies, there have been relatively few studies of sterol biosynthesis in *Acanthamoeba* and those available hampered by the technologies available at the time. Nonetheless there are a number of intriguing aspects of the sterol composition reported in the literature. Firstly, *Acanthamoeba castellanii* (Neff strain) was reported to have 7-dehydrostigmasterol (60%) and ergosterol (40%) as its major sterols, using infrared and ultraviolet spectroscopy gas chromatography and mass spectral analysis^5^. A further study found that *Acanthamoeba culbertsoni* in addition to 7-dehydrostigmasterol and ergosterol also had stigmasta-5,7,22,25-tetraene-3β-ol and stigmasta-5,7,14,22,25-pentaene-3β-ol^15^. The availability of modern GCMS techniques, a microtitre inhibition assay and the recent availability of the *Acanthamoeba* transcriptome project provides an opportunity to address the aforementioned shortcomings in knowledge^10, 16^.

GCMS was first used to determine the major sterol composition of *Acanthamoeba castellanii* (Neff strain) and a recently isolated *A*. *castellanii* clinical T4 isolate described by Mattana *et al*.^17^. The results demonstrate that the sterol composition is conserved between the two strains examined. We find that ergosterol is the major end product of sterol biosynthesis in *Acanthamoeba* in contrast with previously published work that reported the phytosterol 7-dehydrostigmasterol as the major sterols. In fact, we find no evidence of any of the phytosterols, including 7-dehydrostigmasterol, in *Acanthamoeba*. Moreover, using GCMS we have identified a number canonical putative precursors of ergosterol as well as a number of non-canonical precursors. Interrogation of the transcriptome project has allowed us to propose a hypothetical pathway for sterol biosynthesis in *Acanthamoeba* that incorporates the sterols identified by GCMS. This pathway has elements of the canonical pathway, but also contains elaborations that ultimately feed back into the canonical pathway to provide putative alternative routes to ergosterol. Future studies, potentially using labelled precursors will be necessary to test the unique aspects of this candidate pathway. We have interrogated the transcriptome project for *Acanthamoeba* and have identified a set of putative predicted genes that we propose provide the means to produce the identified sterols.

In addition, through testing a number of azoles against a set number of isolates of *A*. *castellanii* and *A*. *polyphaga* we establish that there are relatively small differences between *A*. *castellanii* and *A*. *polyphaga* isolates in their susceptibility to specific azoles. Consistent with this we demonstrate that the assumed target of these azoles 14α**-**demethylase is well conserved between all 5 isolates examined. However, we demonstrate that voriconazole is far superior to the other azoles tested as it inhibits parasite growth earlier (at 24 hours) and at lower concentrations. The superior effects of voriconazole are consistent with a previous report that demonstrated this azole and itraconazole were superior to fluconazole in inhibiting *A*. *castellanii* and *A*. *polyphaga in vitro* ^18^. Importantly, this study also noted that voriconazole and itraconazole had lower dissociation constants for *Acanthamoeba* 14α**-**demethylase than fluconazole^18^. The results from previous studies that found voriconazole to have an IC_50_ of 1.24 µM and 7.16 µM against a variety of *Acanthamoeba* species are also broadly consistent with the ranges found in this study^6, 19^. The fact that voriconazole has also been reported to show efficacy against cysts over a period of 7 days is encouraging^20^.

Although the above studies strongly imply that 14α**-**demethylase is the target of voriconazole in *Acanthamoeba* this had not been formally demonstrated. Furthermore, target validation of this enzyme, or any other for that matter, would normally be achieved through gene-deletion experiments which are not currently possible in *Acanthamoeba* as it is highly polyploid. Thus we employed GCMS to determine the mode of action of voriconazole. Our results demonstrate that voriconazole at IC_50_ levels significantly inhibits the production of ergosterol biosynthesis and the ergosterol intermediate 4,4-dimethyl-cholesta-8,14,24 trienol in *Acanthamoeba*. In addition, lanosterol, the substrate for 14α**-**demethylase and an ergosterol precursor accumulated in voriconazole treated *Acanthmoeaba*. These observations validate 14-α-demethylase as the target of voriconazole.

Overall the multidisciplinary studies described herein, making use of the *Acanthamoeba* transcriptome, analytical GCMS and functional cellular assays yield the most comprehensive description of sterol biosynthesis and composition in *Acanthamoeba* to date and a candidate biosynthetic pathway for future validation. The results demonstrate the potential benefits of testing diverse azoles in *Acanthamoeba* and the GCMS technique described herein provides the means to verify their mode of action in *Acanthamoeba*.

## Materials and Methods

### *Acanthamoeba* strains and culture conditions

Five isolates of *Acanthamoeba* spp. were used for comparative results. An *Acanthamoeba castellanii* of the T4 genotype isolated from a case of *Acanthamoeba* keratitis (referred to *A*. *castellanii* T4 clinical) was donated by Dr. Antonella Mattana (Sassari, Italy). *A*. *castellanii* (Neff) was originally obtained from Dr Keith Vickerman (Glasgow, United Kingdom). *A*. *castellanii* ATCC 50370 and *Acanthamoeba polyphaga* ATCC 50371, American clinical keratitis isolates, were originally obtained from the American Type Culture Collection (ATCC, United States) and *A*. *polyphaga* CCAP 1501/18 was originally obtained from the Culture Collection of Algae and Protozoa (CCAP, United Kingdom).

All strains were maintained by biweekly passages in 10 ml of Protease Peptone-Glucose Media containing Protease Peptone (Sigma, UK), 15 g/litre; glucose (Alfa Aesar, UK), 15 g/litre; Potassium phosphate monobasic (Sigma, UK), 330 mg/litre; Thiamine (Sigma, UK), 1 mg/litre; L-methionine (Sigma, UK), 14.9 mg/litre; B12 (Sigma, UK), 8.33 µg/litre; Biotin (Sigma, UK), 16.66 µg/litre additionally 1 ml/litre of a separate salt solution containing calcium chloride (Sigma, UK), 1.5 g/litre; Iron (III) chloride (Sigma, UK), 200 mg/litre; magnesium sulfate heptahydrate (Sigma, UK), 24.6 g/litre, had to be added separately. The media of isolates Neff, Clinical T4 and ATCC 50370 were supplemented with 100 I.U./mL penicillin and 100 (μg/mL) streptomycin (Sigma, Poole, United Kingdom). Unless stated otherwise all isolates were grown at room temperature in 75 cm^2^ tissue culture flasks (Corning, Amsterdam, The Netherlands).

### GC-MS sample preparation

8 × 10^6^ *Acanthamoeba* were pelleted by centrifugation at 1,200 *g* for 5 minutes. The media was then discarded and 5 ml of a 1/1 chloroform/(90% methanol, 10% water) mix was added. The cells were then transferred to a clean glass vial and left overnight at 4 °C. The cells were homogenised and the lower chloroform containing layer of approximately 2.5 ml was transferred to a clean glass tube. Additional chloroform was then added and the cells homogenised again, this was repeated twice more accounting for a total of 10 ml of chloroform being extracted. The chloroform solvent was then evaporated and the lipids reconstituted with 500 µl of chloroform prior to being analysed.

The presence of steroids was analysed on a Thermo Finnigan Polaris Plus GCMS. Initial temperature was at 100 °C, initial time of 1.00 min, with 1 ramp at a rate of 20.0 deg/min to a final temperature of 320 °C. EIMS is set at detector voltage of 500.0 V with a source temperature of 220 °C while the GC interface temperature is at 220 °C with an emission current of 150 µV. Full scan acquisition is at 2.5 scans per second where the scan range starts at 40 amu to end at 650 amu. Syringe volume is at 10 µl. The column was a Factory Four^TM^ capillary (VF-1ms 30 m × 0.25mm, 0.25 µm). Compounds were identified with NIST online database library.

### Bioinformatical analysis

Genes encoding the enzymes belonging to the sterol synthesis pathway were identified in GenBank™ (http://www.ncbi.nlm.nih.gov/genbank) both by word search (*specific enzyme name* AND *Acanthamoeba*), verified in amoebaDB^21^ and BLASTx for those enzymes not yet specifically identified in the *Acanthamoeba* genome/transcriptome using combination of *Saccharomyces cerevisiae* and *Arabidopsis thaliana* sterol pathway enzymes. The pathway was constructed using the KEGG metabolic pathway (http://www.genome.jp/kegg/pathway/map/map00100.html).

### PCR for 14α**-**demethylase

RNA was extracted and complementary DNA was synthesised as described in ref. 22. Oligonucleotides (5′ CCTCCATCTTCTGAATCTGTCGAGG 3′ and 5′ GGTGTGATTGCTCACGTTCGCCTTC 3′) were designed using PCR primer tool in Genbank and they were synthesised commercially by Life Technologies Ltd. (Paisley, UK). Automated sequencing was performed commercially by Source Bioscience (Nottingham UK).

### Drug Preparation

The majority of the drugs used required individual methods of preparation to ensure that a known concentration was in solution. Econazole (Sigma, UK) was dissolved at 112.43 mM in 1:1 chloroform:methanol solution. This was then diluted in PG media at 1 in 200 to give a stock solution of 562 µM. The preparation of miconazole and sulconazole (all from Sigma, UK) was identical. Drugs were dissolved in DMSO (Sigma, UK), to give a concentration of 208.70 mM for miconazole and 217 mM for sulconazole. These solutions were diluted in PG media to give concentrations of 3.339 mM and 3.472 mM, respectively. Residual drug was solubilised by adjusting the pH with HCl and the final concentration adjusted to 104 µM and 109 µM. Tioconazole (Sigma, UK) was dissolved in DMSO (Sigma, UK) at 500 mM and diluted in PG media to give 16 mM. Residual drug was solubilised by addition of HCL before diluting in PG to give a final concentration of 250 µM. Voriconazole was dissolved in DMSO at 28.63 mM and diluted in PG media to 143 µM. The final formulation of each drug was adjusted where necessary to pH 7.0.

### AlamarBlue Assay

The AlamarBlue, resazurin assay was carried out as previously described^10^. Seeding densities for each of the isolates at each of the time points was determined empirically as previously described. Each drug was tested over a wide range of concentrations for efficacy against the 5 *Acanthamoeba* isolates at 23.3 °C for 24 hours or 96 hours. Six hours prior to the end of the incubation period 10 μl of AlamarBlue reagent (Abd Serotec, UK) was added to all wells except wells that act as a blank. The plate was then incubated for a further six hours at 23.3 °C in the dark. Controls consisted of a media blank, trophozoites with media alone, trophozoites with the highest concentration of solvent found in the drug samples, trophozoites with 12.5 μM chlorhexidine and media with the highest concentration of drug alone. The final volume for all wells was 100 μl.
